# m-Path: an easy-to-use and highly tailorable platform for ecological momentary assessment and intervention in behavioral research and clinical practice

**DOI:** 10.3389/fdgth.2023.1182175

**Published:** 2023-10-18

**Authors:** Merijn Mestdagh, Stijn Verdonck, Maarten Piot, Koen Niemeijer, Ghijs Kilani, Francis Tuerlinckx, Peter Kuppens, Egon Dejonckheere

**Affiliations:** ^1^ Faculty of Psychology and Educational Sciences, KU Leuven, Leuven, Belgium; ^2^ Department of Medical and Clinical Psychology, Tilburg University, Tilburg, Netherlands

**Keywords:** daily life, smartphones, experience sampling, ambulatory assessment, mobile sensing, blended care, just-in-time adaptive interventions, mental health

## Abstract

In this paper, we present m-Path (www.m-Path.io), an online platform that provides an easy-to-use and highly tailorable framework for implementing smartphone-based ecological momentary assessment (EMA) and intervention (EMI) in both research and clinical practice in the context of blended care. Because real-time monitoring and intervention in people's everyday lives have unparalleled benefits compared to traditional data collection techniques (e.g., retrospective surveys or lab-based experiments), EMA and EMI have become popular in recent years. Although a surge in the use of these methods has led to a myriad of EMA and EMI applications, many existing platforms only focus on a single aspect of daily life data collection (e.g., assessment vs. intervention, active self-report vs. passive mobile sensing, research-dedicated vs. clinically-oriented tools). With m-Path, we aim to integrate all of these facets into a single platform, as it is exactly this all-in-one approach that fosters the clinical utility of accumulated scientific knowledge. To this end, we offer a comprehensive platform to set up complex and highly adjustable EMA and EMI designs with advanced functionalities, using an intuitive point-and click web interface that is accessible for researchers and clinicians with limited programming skills. We discuss the strengths of daily life data collection and intervention in general and m-Path in particular. We describe the regular workflow to set up an EMA or EMI design within the m-Path framework, and summarize both the basic functionalities and more advanced features of our software.

## Introduction

Psychological phenomena, such as mood, thoughts and behavior, are always embedded in a specific context, and we can only fully grasp their meaning when we also consider the circumstances and situations in which they occur (1). Recent advances in mobile technology have made this situated notion generally accepted, and have allowed researchers and healthcare practitioners to monitor and modify people's feelings, appraisals and actions exactly where they take place: in daily life (2, 3). In this paper, we aim to further stimulate these practices by introducing m-Path, an easy-to-use and highly tailorable platform for daily life data collections. m-Path is a tool designed for application in both research and clinical practice. Yet, given the (mostly research-focused) readership of this article, we will focus on the research implementation in the first place. However, all features and functionalities may equally be used by professionals in clinical practice.

## The strengths of ecological momentary assessment and intervention

Although standardized lab experiments and cross-sectional survey research have been proven invaluable in uncovering the basic processes and building blocks of the human psyche, psychological researchers have long been aware of the limits of these traditional data collection methods (4, 5). Lab experiments are often criticized for their nomothetic or between-person focus and restricted generalizability to everyday contexts (6, 7). In a similar vein, self-report surveys that invite participants to rate a series of general statements about their person, or to provide an aggregated estimate of past experiences, typically suffer from response or memory biases that severely undermine the validity of these measures (8).

Many of these critical challenges (e.g., the predominant between-person focus of these methods, their artificial and retrospective nature) are effectively remedied in Ecological Momentary Assessment [EMA, also known as Experience Sampling Methods (9, 10)] and Intervention [EMI (11)] research, where the idiographic, ecological and momentary qualities of collected data are the primary pursuit (12, 13). In EMA studies, participants repeatedly report about their momentary behavior, feelings, thoughts and context as they live their lives (2). Not only does this provide researchers with a particularly natural window into people's daily routine, it allows them to understand the typical time course of these personal experiences, their real-life antecedents and consequences, and within-person interrelations (14). Likewise, in EMI studies, the idea is to alter certain behaviors or experiences in everyday life via proposed exercises or interventions (15). Besides evaluating the general efficacy of (real-life) manipulations under natural circumstances, EMI's personalized focus enables researchers to determine what strategy works for whom, when and in what dose (16).

As mentioned earlier, many of these benefits are of similar importance in clinical practice (17). In the context of eHealth, mHealth or blended care [i.e., combining face-to-face therapy with online tools (18)], clinicians increasingly rely on EMA applications to obtain better insight into the symptoms, context, their contingencies, and general daily functioning of their patients [e.g., routine outcome monitoring (19)], and they may even add EMI modules to intervene in everyday life [e.g., just-in-time adaptive interventions (20)].

## The strengths of smartphone-based EMA and EMI studies

The rising ubiquity of mobile technology (21) has facilitated the adoption of EMA and EMI in behavioral research and clinical practice (22). Traditionally, participants were prompted at predefined moments *via* a programmable wristwatch or palmtop with the instruction to complete an EMA diary with paper and pencil (23). Although this paper-and-pencil approach involved little costs, and participants were generally well-acquainted with the method, collected data could easily get lost, and data entry was a labor-intensive and error-prone process. Critically, written diaries could not prevent the problematic phenomenon of *back-filling*, where participants failed to complete the momentary EMA surveys at the scheduled time, but retrospectively filled out their paper diaries about earlier experiences later in time (24).

Today, paper EMA booklets have largely made way for digital entries via smartphones or other electronic devices, adequately overcoming some of the initial shortcomings of historic EMA research (14). Inviting participants to download an EMA app on their personal smartphone allows researchers to signal and survey with the same device. The use of digital timestamps reveals exactly when participants initiated and completed momentary EMA questionnaires, eliminating the possibility of back-filled EMA responses. An online connection allows all EMA responses to be stored automatically to the database without mistakes, and makes sure that the data is backed-up regularly in case of phone loss or malfunctioning (25).

But smartphone-based EMA studies also hold other new promising opportunities. Besides participants' commitment to actively rate their momentary subjective experiences in an app, these self-report data may be effectively complemented with passive mobile sensing (26–28). Relying on the embedded sensors in people's smartphones (e.g., GPS, Bluetooth, pedometer, etc.), researchers are able to unobtrusively acquire objective information about participants' current surroundings (e.g., location, noise, etc.) and behavior (e.g., social interaction, activity level, etc.) (29). Relatedly, an app-based EMA environment has advanced the possibility to extend basic assessment with periodic EMI reminders, automated supportive messages or therapeutic exercises, creating a set-up that is more interactive and engaging (22). Finally, the online setting allows researchers to set up an EMA study entirely remotely, without participants needing to attend physical lab meetings. This is particularly convenient when conducting EMA or EMI research with difficult-to-access study populations [e.g., migrants (30)] or in exceptional conditions [e.g., solitary confinement during a pandemic (31)]. For studies in progress, an online connection continuously provides real-time information about participants' EMA and EMI interactions, allowing researchers to closely monitor people's study compliance (32).

## The strengths of m-Path

Because the added value of daily life research is evident, and the embracement of phone-based EMA and EMI applications is quickly progressing, the number of available tools, platforms and software solutions is taking a similar lift-off^1^. However, many existing applications are primarily designed to address a single aspect of data collection in daily life. If we organize the current landscape of available tools, we could dissect three primary bipolar dimensions that shape the field of mobile technology: (a) tools for scientific research vs. clinical (blended) care, (b) applications for assessment vs. intervention, and (c) platforms that collect active self-report vs. passive (mobile) sensing data. Very few (if any) existing platforms offer software services for all of these poles. However, an all-in-one solution, where each of these facets is integrated into a single platform, could foster the clinical utility of accumulated scientific knowledge. To fill this gap, we introduce m-Path (www.m-Path.io), an online web-platform that supplies behavioral researchers and clinicians with an intuitive and flexible framework to conduct smartphone-based EMA and EMI studies, focusing on both active and passive data streams.

Originally, m-Path's precursor, mobileQ (33), only offered an open-source (self-report) EMA platform for researchers, that was moreover only compatible with Android phones. However, with the increasing uptake and demonstrated effectivity of a blended care approach in clinical practice (34) and the rapid accumulation of mobile sensing research (28), m-Path aimed to broaden this focus, providing EMA as well as EMI services, to both researchers and clinicians, for the collection of both active self-report and passive mobile sensing data, on Android and iOS smartphones.

In the following paragraphs, we dissect five key strengths that make m-Path a useful tool for research and/or therapy. We discuss how m-Path is (a) easy to use, while simultaneously providing (b) comprehensive EMA and EMI solutions that are (c) highly tailorable. We explain how our platform (d) facilitates the direct translation of evidence-based research applications to clinical practice, and (e) how we ensure the secure and anonymous storage and processing of sensitive participant and/or patient data (see Table 1 for a summary).

**Table 1:** Overview of m-Path's core strengths and some non-exhaustive examples.

Strengths	Examples
1. Easy-to-use	- Point-and-click web interface - No coding skills required - Extensive manual and online helpdesk - Real-time try-out of surveys or interventions in emulator or on personal device
2. Comprehensive	- EMA: active (self-report) and passive (mobile sensing) assessment - EMI: just-in-time adaptive interventions and stand-alone applets - Participants: study single individuals, dyads or system interactions - Gamification options: progress bar, reward system, feedback, etc. - Devices: suitable for both iOS and Android phones
3. Tailorable	- Survey content: vast collection of item types and multimedia that can be customized further - Survey flow: randomization, conditional logic, piped text, real-time computations, etc. - Study flow: event- and time-based sampling, reminders, follow-ups, expiration windows, etc.
4. Translational	- Select (validated) surveys from the public m-Path library or share personal ones within closed contexts - Host (evidenced-based) interventions or applets and share them directly with our community of practitioners - Participants and patients can share data with multiple researchers or clinicians
5. Privacy and security	- No contact information (name or e-mail) is required for participants or patients to subscribe to m-Path - Private alias or nickname is used to identify participants or patients - Server-phone communication is real-time and end-to-end encrypted - Recovery codes are available to restore data connections - m-Path is GDPR compliant - The online dashboard allows for two-factor authentication

### Easy-to-use

First, because clinical practitioners comprise a sizeable part of our user population, m-Path is built on the premise to create an easy and intuitive user experience when designing an EMA or EMI study in our framework. While many existing EMA and EMI tools require considerable coding skills or the familiarization with difficult programming languages, m-Path users set up their study in a simple point-and-click web interface. A shallow learning curve allows researchers to learn about all m-Path functionalities on the job, and makes our software accessible for people who may be less technologically experienced. In case users do run into some difficulties, accessing our extensive manual (www.m-Path.io/manual) or online helpdesk (www.reddit.com/r/mpath) may remediate their problem.

### Comprehensive

Second, m-Path pursues a complete solution for daily life research, offering a wide and diverse range of features and possibilities without compromising on quality. For example, for EMA researchers, m-Path can be used to collect subjective self-report data, as well objective context information via mobile sensing. Beyond single participants, researchers can study dyads or systems interactions, where subjects may repeatedly respond to the momentary answers of other individuals. Next, EMI researchers may integrate EMA input into their design to provide participants with dynamical exercises or adaptive interventions, or they may create stand-alone (self-help) applets with instructions or multi-media content for people to consult at their own convenience. In addition, our platform includes optional features like progress bars and an award-system to improve response rates or to stimulate certain behaviors based on principles of ethical gamification (35), or the presentation of real-time feedback for participants (personal time series in the app) and researchers (basic analyses in the online dashboard). In the section “Advanced Functionalities”, we provide a more elaborate discussion on some of these features. Finally, for participants, the m-Path app runs on both Android and iOS smartphones.

### Highly tailorable

Third, m-Path allows EMA and EMI researchers to extensively customize a study following their own specific research needs. Regarding survey or applet flow, researchers can select from a wide array of item types. Advanced conditional logic enables branching within the questionnaire or applet, and containers can be used to set up randomizations or to display multiple items on one page. Previous answers can be recycled with piped text, within and across different interactions, or may serve as the input for real-time (phone-based) computations (e.g., to calculate an average of two ratings). Regarding survey or applet lay-out, every item can be edited further and tailored according to the researcher's personal preferences. Researchers can use formatted text, edit an item's anchors, qualitative labels or associated numerical values, allow participants to see their previous response or to skip the question. Finally, regarding sampling schemes, researchers can schedule EMA or EMI interactions in an event-contingent [i.e., initiated upon participants' request via a button or in a stand-alone applet (36)] or signal-contingent way [i.e., beyond participants' control at predefined time points (3)]. In the latter case, researchers can consult the calendar to schedule fixed signal moments (e.g., exactly at noon) or random interaction intervals [e.g., somewhere between 2 and 4 PM (25)]. For each prompt, reminders and expiration windows can be introduced. Together, these tailorable functionalities enable researchers to move away from m-Path's default settings, creating a complex and highly customized EMA or EMI research design.

### Translational

Fourth, m-Path consolidates connection between all users of the platform by facilitating the exchange of materials, schedules or entire study designs. Within private contexts (e.g., project groups or research departments) researchers can share, mutually revise and adapt all EMA surveys or EMI exercises for their study. More broadly, researchers may browse and select materials from the public m-Path library, or upload personally created content for other members of the community to use (e.g., validated questionnaires or applets). Since m-Path is also adopted by clinicians and mental health practitioners, our platform maximizes the translational value of evidence-based research applications. In each phase, researchers have full control over the development process, from design to effectivity research to the direct distribution of their product to actual practitioners, allowing for a smooth reiterative process of refinement and adaptation without having to rely on external software developers. Finally, also participants themselves have the possibility to share their data with m-Path registered therapists or clinicians.

### Private and secure

Finally, m-Path has been developed according to the privacy-by-design principle (37). When registering to m-Path, participants or patients are not required to provide identifiable (contact) information, such as a name, e-mail address or phone number. By simply specifying an alias or a nickname when subscribing (e.g., an agreed participant ID), all collected EMA or EMI data is processed and stored anonymously. Albeit slightly more cumbersome compared to standard restore strategies that are user identifiable, a unique recovery code is provided to prevent participant-data disconnections in the case of phone malfunctioning or device changes. In line with the General Data Protection Regulation (GDPR) of the European Union, participants have full control over their data, meaning that they can delete their data, block researchers' access, or share their data with other people (38). Phone-server communication is end-to-end encrypted, and data back-ups occur instantly after each participant interaction to prevent missing data in the case of phone loss or damage. The online researchers’ dashboard allows for two-factor authentication. For m-Path's source code, we make use of code obfuscation, which results in machine code that is almost impossible to unravel for external programmers (i.e., code obfuscation conceals the logic and purpose of the programming code, making m-Path difficult to reverse engineer). This should further protect us from the discovery of unforeseen vulnerabilities in our tool.

## Designing an EMA or EMI study in m-Path

In the next paragraphs, we review m-Path's general workflow, explaining the most essential steps in setting up and conducting an EMA or EMI study. This is followed by a more in-depth discussion of some of the more advanced features in the m-Path framework. However, to have a complete overview of m-Path's features and to stay up to date with the latest functionalities, we refer the reader to our online manual (www.m-Path.io/manual)^2^.

### The m-Path workflow

**Figure 1:**
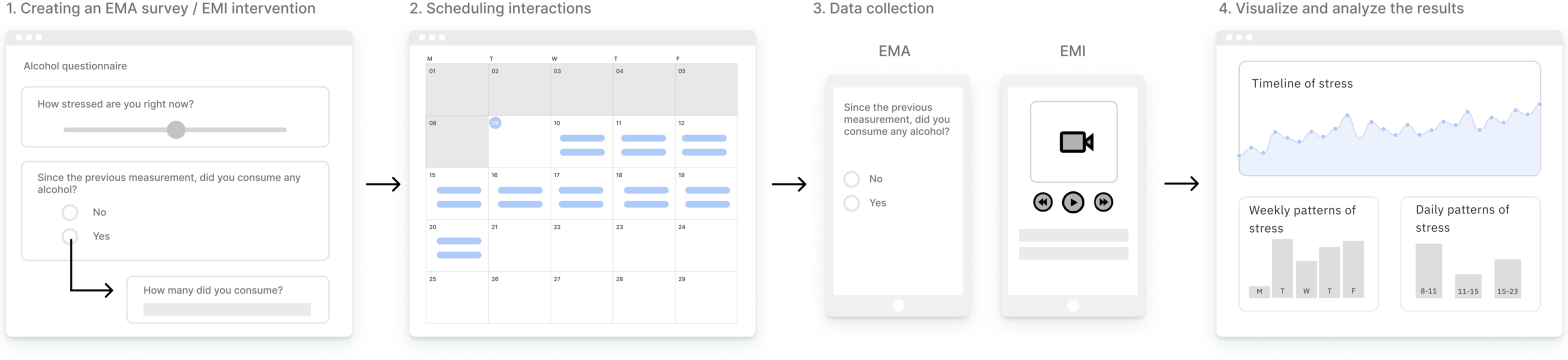
The m-Path workflow. First, researchers create and fine-tune their EMA survey or EMI intervention in the interaction editor (Panel 1). Next, these EMA or EMI interactions are scheduled in the calendar view (Panel 2). Third, participants receive notifications on their personal smartphone to interact with the EMA or EMI content (Panel 3). Finally, researchers can analyze incoming results of a single participant real-time *via* modifiable graphs and charts in the online dashboard (Panel 4).

Researchers can access m-Path's online dashboard at www.m-Path.io/dashboard. After logging in with an existing account or having created a new one (always linked to a personal e-mail address), researchers get access to their study workspace. At first, this space will be empty, but once the study is active, enrolled participants will be located here, together with information about their alias or nickname, time of subscription and real-time response rate. For now, researchers should create a “Protocol”, a kind of EMA or EMI study template to apply to future participants. For a general infographic of the m-Path workflow, we refer to Figure 1.

#### Designing and scheduling an interaction

Under the tab “Create”, researchers may devise and schedule a new EMA or EMI interaction for their “Protocol”. In a pop-up window, researchers need to specify whether they prefer to start from scratch or would like to adapt an existing template from a personal or public library. Multiple interactions can be designed simultaneously (e.g., a separate morning and evening EMA questionnaire, an EMA survey combined with an EMI intervention, etc.), and the properties of each interaction can be customized under their respective heading (e.g., the type of interaction, their notification settings, etc.). After assigning their personal smartphone as the desired test device in the general tab “Settings” (we will cover the app installation and connection to the study later), researchers can immediately try out the flow of created interactions on their own smartphone via the button “Test on me”, similar to the way a participant would experience it.

#### Creating an EMA survey

The flow of an EMA questionnaire is represented from the top down in the m-Path dashboard (see Panel 1 in Figure 1). Researchers should simply start typing the first question into the search bar or enter the name of a specific question type. The list of question types is extensive, covering both some commonly used survey items (e.g., ordinary text instructions, open-ended questions, Likert and visual slider scales, yes/no, single and multiple choice questions, etc.), as well as more advanced question types (e.g., order options, interactive smiley questions, indicating body parts, etc.). All items have both shared (e.g., allow skip) and unique (e.g., customize slider anchors) settings that can be edited further in the sidebar next to an item. Changing the label of a specific question will help researchers to identify each variable in the data output and analysis dashboard. Each item can be previewed separately in an online emulator by clicking the eye icon next to the question.

Two additional features deserve further explanation. First, clicking the downward arrow that precedes a particular item, creates the branch of a conditional structure, visually represented by a slight indent of the subsequent items (see Panel 1 in Figure 1). The conditional logic can be graphically specified in the configuration menu of the contingent item, only triggering this particular (branch of) question(s) when a certain condition in the previous item is satisfied (e.g., only when the numerical rating of a slider exceeds 50, only when the category “other” is selected in a multiple choice question, etc.). There is no limit on the number of conditional levels. Adding subsequent questions to a previous higher level again, allows the flow of a survey to continue once the branch is completed or when the associated condition was not met. Second, container items can be used to group particular questions together. This can come in handy when researchers wish to display multiple questions on a single screen (without the need to press “Next”) or when they would like to randomize the order in which a series of items is presented. Together, these functionalities allow researchers to design versatile and complex survey flows.

#### Creating an EMI intervention or applet

Although EMI interactions may feel qualitatively different, their set-up is similar to an EMA interaction. In the case of signal-contingent interactions, EMI interventions can be embedded in an active EMA survey (as a conditional branch; e.g., only triggered when participants' current *sadness* levels exceed 80 on a visual slider), they can be triggered later in a delayed follow-up prompt, or they can function as entirely independent prompts. In terms of content, participants can interact with a series of written instructions, images (image-URL), audio (.mp3-URL) or video (YouTube-URL) files. Each medium can be stored locally on the smartphone, except film clips, which require an active internet connection.

In the case of a stand-alone applet within the m-Path application, researchers can set up a conditional structure with multiple button items placed in a (homepage) container to create a modular feel. The general lay-out of the homepage and its buttons can be determined by placing containers within containers and by using the functions “Display on one page” and “Display as row if possible”. By inserting divider items with adjustable width and height researchers may further fine-tune the look of a one-paged container. Assigning custom images to the buttons further personalizes the applet, and under each button researchers can again store different types of (multimedia) content or survey items to interact with. Concluding each modular branch with a command item allows participants to move freely between the different levels of the app. Ticking the “Save and flush” box in the settings of this command item will save the data on all the interactions participants had with the underlying content.

#### Scheduling interactions

Once all EMA or EMI interactions are created, researchers may schedule these in the calendar below (see Panel 2 in Figure 1). To schedule different types of interactions, simply switch between the different interaction tabs. By clicking at a particular location in the calendar the selected interaction is added at that position. Alternatively, researchers can use the automatic “Fill” option to immediately create an entire schedule, or they can copy and extend a particular day or week. For each individual prompt, the properties may be further edited by clicking the scheduled interaction (e.g., set as fixed or random, add reminders or expiration periods, etc.). Once finalized, researchers can anchor the generic schedule of their “Protocol” template to concrete starting dates for actual participants.

Finally, in event-contingent EMA or EMI designs, researchers can also attach an interaction to a button in the home screen of the m-Path smartphone app. Each time participants push that button the interaction will automatically initiate.

#### App installation and data collection

Once researchers have finished the creation of their EMA or EMI template, they can enroll participants to their study. To this end, participants download the m-Path app on their personal smartphone from the Google Play (Android) or App Store (iOS). When launching the app for the first time, it is important that they grant the app permission to send notifications in order to actually receive the EMA or EMI prompts (permission is given by default on Android phones). As mentioned earlier, m-Path strongly advises participants to specify an alias or nickname in order to guarantee their anonymity, and no identifiable information is collected. After reading and agreeing to the general terms and conditions (for more information, see the section “Proprietorship, Product Support, Interoperability and Terms of Use”), a unique recovery code is presented to restore their account (and the associated data) in case of phone loss or app malfunctioning. It is advised that researchers remind participants to save this code elsewhere than on their personal device (e.g., write it down on a private piece of paper). Finally, participants enter the name of the researcher or the invitation code they received to subscribe to the study.

Participants are automatically added to the researcher's study workspace. Although researchers can assign or set up the EMA or EMI template for each individual participant separately, we advise them to select the appropriate “Protocol” as the default baseline setting (see “Settings” > “Auto Enrollment”). By simply ticking the corresponding box, m-Path will automatically allocate the selected template to all newly enrolled participants. Finally, under the same “Auto Enrollment” tab, researchers can also plan an intake interaction (see "Intake settings"). This could be an informed consent or cross-sectional baseline survey that automatically launches a single time when participants subscribe to the study, prior to the start of the actual EMA or EMI study.

During an ongoing EMA or EMI study, participants will receive push notifications when the phone has an active internet connection (see Panel 3 in Figure 1)^3^. However, the app does not need mobile internet to open the notification to present the associated content to participants. All answers and interactions are saved when the app is offline, and sent back to the server once the phone is online again. For standard EMA or EMI interactions, the amount of internet traffic necessary is minimal, as only a small amount of text information needs to be transferred between smartphone and server. When EMA or EMI content includes media (e.g., pictures or audio files), these files need to be downloaded to the app once, prior to the first interaction.

#### Visualize and analyze the results

Finally, in the tab “Visualize”, researchers or clinicians can inspect the real-time results of a single subject (see Panel 4 in Figure 1). In addition to the raw time series (either represented in a graph or downloadable data table), m-Path summarizes the data in the form of basic frequency analyses, linear analyses, week overviews, and time range comparisons. Each graph is interactive, and can be further modified by adding or removing other variables.

These automatic visualizations are primarily designed for clinicians who wish to track patients' complaints in daily life or who want to evaluate the effectiveness of their (real-life) therapeutic interventions (e.g., just-in-time adaptive interventions or routine outcome monitoring). In particular, the export functionality may be useful to enrich existing patient health records with m-Path-generated graphs or charts. For researchers, this tab supports the real-time monitoring of participants' EMA or EMI compliance once data collection has begun, or the automatic creation of feedback graphs as a reward for people's participation in the study. Finally, by default, participants can also track their personal responses over time in the m-Path smartphone app, or they may create an m-Path dashboard login to interact with the data graphs themselves.

Finally, when the study is terminated, researchers can export the data of all participants at once in the “Settings” tab (in .xlsx format). A selection of exported interactions can be specified, and researchers may select whether they want to additionally download order data (i.e., the ordinal sequence in which items were presented), skipped data (i.e., binary file indicating whether items were deliberately left unanswered) or response time data (i.e., completion time per item in milliseconds).

### Advanced functionalities

In this section, we discuss some of m-Path's more advanced features. These entail some functionalities that are not always offered or supported by typical EMA or EMI platforms.

#### Award system

m-Path offers daily life researchers the possibility to rely on a built-in award system that gamifies their EMA or EMI interactions. If this option is selected, participants receive an in-app notification that they received an award when they reached a predetermined goal. Researchers can choose from a set of pre-built default awards (e.g., completing all EMA surveys for that day) or they can create custom awards specifically for their study. Participants can be rewarded for the frequency of their interactions (e.g., performing a streak of 15 uninterrupted EMI exercises), but also for the content of their responses (e.g., indicating that they did not consume any alcoholic beverages). All acquired and locked achievements are displayed in the Awards menu of the m-Path app, and each award can be fully tailored with a unique icon, text, color, set of progress options and fireworks.

#### Real-time computations

The advanced computation item allows researchers to perform real-time phone-based mathematical computations during the completion of an EMA survey or EMI interaction. Computations are performed in the background, and are based on the labels of previously presented items. Their mathematical expression can be very basic (e.g., calculate the average of five positive mood items) to quite complex (e.g., only trigger the conditional EMI exercise when the two negative emotions ratings are all higher than the two positive ones). We implemented the use of reverse scores, logical expressions and a random number generator. There is also the possibility to recycle answers from a previous assessment using the “load and save” functionality.

#### Follow-up items

A follow-up item can be used to automatically trigger a contingent EMA or EMI interaction later in time, based on participants' current responses. Researchers can specify a fixed delay (e.g., 5 min later) or a random delay interval (e.g., somewhere between 5 and 10 min after completion of the first interaction). This feature can come in handy when planning so-called measurement burst designs (39), where multiple follow-up interactions are automatically scheduled when participants' indicate that a certain event or behavior is taking place (40). Similarly, this item can be used in the context of statistical process control (41), where the assessment frequency of a momentary EMI or EMA interaction is temporarily increased when a psychological phenomenon exceeds a predefined normative boundary.

#### Passive mobile sensing

Smartphones have a wide range of sensors and software that automatically collect data on (changes in) users' phone usage and location, providing researchers and practitioners with a unique chance to include objective information about people's context and behavior in everyday life (27). Furthermore, because mobile sensing is continuous and unobtrusive, it allows for a far faster and less burdensome tracking than typical self-report (26). Together, these promises have piqued people's interest in passive sensing data, ushering in a new era of *digital phenotyping* in behavioural and clinical research (42).

In this spirit, we developed m-Path Sense (43), a special version of our m-Path app for researchers who wish to collect both mobile sensing and self-report EMA data. To collect these passive data, we implemented the Copenhagen Centre for Health Technology (CACHET) Research Platform [CARP (44)] into m-Path, a highly extensible open-source cross-platform for mobile sensing (43). Table 2 lists the various types of sensing data that m-Path Sense can collect, both for Android and iOS devices.

**Table 2:** Overview of sensing data that can be collected with m-path sense.

Type of data	Description	Android	iOS
1. Ambient noise	Volume (in decibel) of ambient noise	Yes if app in foreground	Yes if app in foreground
2. App usage	Usage time per app	Yes	No
3. Bluetooth	Information about Bluetooth devices in vicinity	Yes	Yes if app in foreground
5. Connectivity	Wi-Fi ID (encrypted), connected to Wi-Fi or mobile data	Yes	Yes
6. Device	Information about the device	Yes	Yes
7. Location	GPS coordinates every minute. These are stored encrypted	Yes	Yes
8. Pedometer	Number of steps according to the phone's pedometer	Yes	Yes
9. Physical activity	Number of minutes walking, cycling, driving, etc.	Yes	Yes
10. Screen activity	Time of screen on/off/unlocked events	Yes	No
11. Sensor data	Accelerometer, gyroscope (rotation) and light sensor	Yes	No light
12. Weather	Weather from the internet based on current location	Yes	Yes

The processing and storage of passive sensing data takes place via a separate app that runs in the background on participants' devices and exists entirely independent from the original m-Path platform to ensure participants' full privacy. Similar to the original m-Path app, m-Path Sense is GDPR compliant, and participants have full control over their data. m-Path Sense comes with an R-package to automatically read all collected data into a SQLite database, and a straightforward web-based Shiny dashboard (45) to monitor all sensor data being transferred from participants' phones to the server in real time (e.g., the visualization of raw time series for each mobile sensor, the amount of data collected per sensor within a certain time interval, etc.). This is particularly beneficial to ensure that the application does not unexpectedly quit (as explained in Footnote 3, phones tend to automatically kill background apps for battery optimisation purposes), or to catch and remediate potential problems during data collection. To integrate the passive sensing data with the active self-report data, users can merge both data outputs on the basis of the time stamp variable that is identical in both files. The sensing of other external mobile devices (e.g., activity trackers or smartwatches), as well as the real-time interaction between active and passive data streams (e.g., EMA self-reports that are triggered by passive sensing data) are currently not in place, but these are promising avenues that we are looking into for future development.

## Proprietorship, product support, interoperability and terms of use

m-Path is currently free to use in its basic form, but is a paying service when adopting additional premium features, seeking extensive (priority) support, or requesting customized workshops (see www.m-path.io/landing/pricing/ for an up-to-date pricing page). This revenue is used for further development, system maintenance and support, and legal consultancy (e.g., GDPR and Medical Device Regulation). At this point in time, m-Path is being developed, maintained and hosted by the KU Leuven. The m-Path servers are located in Leuven and Heverlee, Belgium, with entrance to the servers being limited to the personnel who maintain the infrastructure. Upon request, self-storage of data is possible when paying a hosting fee.

When certain desired features or functionalities are currently not supported by our platform, researchers may reach out to our development team via the contact form on our website, after which we provide them with a custom offer. m-Path will be supported in the foreseeable future (e.g., highlighted by various multi-year contracts with some of our customers), either by the university itself or a spin-off company. m-Path is currently closed-source for security purposes, but is open to automated API-based interoperability requests from other care systems or electronic health records. All legal documents, including the terms and conditions, license agreement and privacy statement (as per GDPR), can be found on our website (see www.m-path.io/LegalPage/legal.html).

## Performance and previous applications

m-Path already has a broad and active user base. Up until now, more than two million questionnaires, sent by more than 3,500 unique researchers and practitioners around the world, have been answered by more than 50,000 participants. The large-scale adoption of our platform prior to the publication of this article permitted us to extensively pilot the software (e.g., code debugging, feature testing, scalability optimization, etc.), evaluate its performance under different circumstances (e.g., various time zones, device types, study designs, etc.), and improve the usability of both the app and online dashboard (e.g., via self-constructed UX surveys, think aloud protocols or qualitative interviews). Given that all processed and saved data are purely text-based, minimal working and long-term storage requirements allow us to effectively scale up our system when the user base or traffic on our platform continues to grow.

Regarding specific study designs, m-Path has been successfully used to conduct conventional short-term EMA studies (both active and passive) and EMI studies, long-term single-case EMA studies, event-contingent EMA measurement burst designs, within- and between-person experimental EMA manipulations, dyadic and family EMA studies, and stand-alone EMI RCTs. Regarding study populations, m-Path has been instrumental in data collections with different age groups (from high-school adolescents, to university adults, to elderly, etc.), various hospitalized patient groups (with both mental and physical conditions), disabled individuals, and large-scale community samples. Finally, m-Path has been used to address various types of research questions, such as investigating the temporal dynamics of psychological phenomena (e.g., by means of auto-regressive modelling or statistical process control to determine changes in mood, psychological or physical complaints, sleep quality, social relations, etc.), the within-person interrelations of psychological phenomena [and between-person differences therein; e.g., by means of (multilevel) vector auto-regressive modelling, network analysis, etc.], the situational contingencies of these constructs (e.g., by means of survival analysis to evaluate the short-term or long-term psychological impact of events, etc.), and the effectiveness of EMI applications (e.g., RCTs of a mindfulness app or an app to promote healthy eating behavior, etc.).

Finally, quality evaluations indicate that our software is well received among our users. In a recent self-conducted survey, dashboard users (i.e., researchers and/or clinicians; *n* = 27) indicated that they would highly recommend m-Path to others (6.37 on a scale from 1 to 7, *SD* = 0.74).

## Technical background

m-Path's mobile app is designed in Flutter, an open-source user-interface framework provided by Google to design multi-platform applications from a single codebase. This software allows the flexible deployment to multiple devices (and screens) that run on various operating systems (both iOS and Android). To push notifications to user's mobile phones, m-Path uses Firebase Cloud Messaging, a Google cross-platform notification system that allows instant communication between server and phone^4^. This external service only requires and receives information about the time and date of sent notifications, it has no access to the actual survey content or responses of the associated questionnaire.

**Figure 2:**
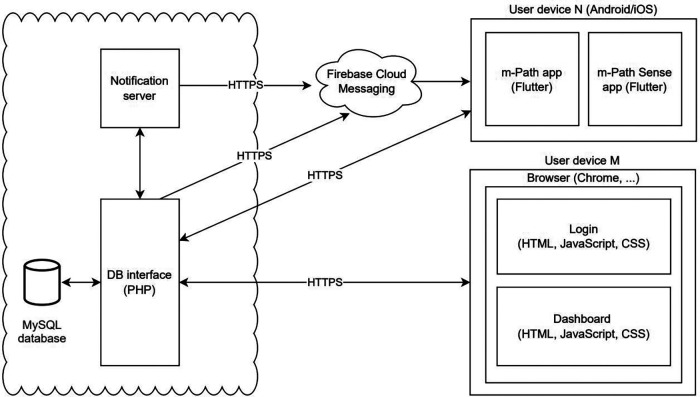
Overview of m-Path's technical components (with main programming languages between brackets) and third-party services. Participants/patients interact with one of the mobile m-Path applications installed on their Android / iOS device. The regular app is used for standard EMA or EMI, m-Path sense supports passive mobile sensing. Researchers/clinicians design their studies and analyze incomming results in a web-based dashboard. We use Firebase Cloud messaging as an external service to push notifications.

For m-Path's front-end, we rely on HTML, CSS and JavaScript as our main languages to design the web-based dashboard interface. For our system's back-end, we use PHP as a scripting language to interact with a MySQL database in which we store all scheduled and incoming data. This is an open-source relational database management system that organizes all incoming and outgoing data into one or more data tables that relate to one another. Also in the back-end, there is a continuously running process written in the Julia programming language that tracks all schedules and sends push notifications using Firebase Cloud Messaging (this process is referred to as the Notification server). For a summary of the main components of the system, we refer to Figure 2.

## Conclusion

With m-Path, we hope to offer daily life researchers an all-inclusive software solution when creating and conducting EMA or EMI studies, or for its implementation in clinical practice. Balancing an intuitive user experience with highly customizable features, m-Path users can choose to readily set up a basic study in a few clicks, or to work out quite advanced and ingenious designs, carefully tailored to their very specific research question or clinical needs. In this way, m-Path helps both novice and experienced EMA or EMI adepts to push the boundaries of daily life research and blended care, allowing them to explore uncharted territory when it comes to the natural occurrence of psychological phenomena. In compliance with the reporting guidelines on mobile health software (47), Table 3 explicitly summarizes the standardized *mHealth Evidence Reporting and Assessment* (mERA) criteria that were covered throughout the paper.

**Table 3:** mHealth evidence reporting and assessment (mERA) checklist.

Criterium	Explanation
1. Infrastructure	Participants/patients need a smartphone (iOS or Android) with active internet connection. Researchers/clinicians need a computer with internet access to create and schedule EMA or EMI interactions, and to visualize and analyse results in the online dashboard.
2. Technology platform	m-Path is a closed-source EMA and EMI platform, that relies on Firebase Cloud Messaging for computer-phone communication. The mobile app is designed in Flutter, the online dashboard in HTML, CSS and JavaScript. The server side interface with the MySQL database is written in PHP. The continuously running process that tracks all schedules and sends push notifications using Firebase Cloud Messaging is written in Julia.
3. Interoperability	API-based interoperability connections can be developed to interact with other care systems or electronic health records.
4. Interaction delivery	EMA or EMI interactions can be scheduled *a priori* and sent in a notification-based manner or user-initiated. Collected data may consist of active self-reports (text responses, numerical ratings, photos, voice recordings), passive mobile sensing logs (see Table 2 for specifics), and/or meta-information (reaction times, skipped items, presentation order).
5. Interaction content	m-Path only enables communication between participants/patients and researchers/clinicians. The actual content and responses are provided by the users of our platform. All created materials can be stored locally, shared with others privately or publicly *via* the public m-Path library.
6. Usability testing	The input from various self-created UX surveys, think aloud protocols, and qualitative interviews was used to develop and improve the usability of our online dashboard.
7. User feedback	A self-conducted survey among our dashboard users indicated that they would highly recommend m-Path to others (6.37 on a scale from 1 to 7).
8. Access of individual participants	Any owner of an iOS or Android device can install our mobile app, except for participants/patients with a recent Huawei phone. Active mobile internet or Wi-Fi is required to receive notifications or to upload responses to the server. m-Path supports various accessibility features for those with visual or cognitive impairments (e.g., audio assistance, increased font sizes, smiley ratings or image choice items, etc.).
9. Costs	In its basic form, m-Path is currently free to use. To make use of premium features (e.g., automation, advanced data exports, priority support, etc.), an annual subscription fee is requested (see www.m-path.io/landing/pricing/ for an up-to-date pricing page). All revenues are used for further development, system maintenance and support, and legal consultancy.
10. Programme entry	Anyone who registers with a valid email-address can make a researcher/clinician account to set up an EMA or EMI study in the web-based dashboard. Participants/patients need a study/clinician connection before they can take part in a specific EMA or EMI protocol (either *via* manual lookup or invitation code).
11. Conditions for delivery at scale	Our servers only process and store text-based data. This requires minimal working and long-term storage capacity, which allows us to effectively scale up our system when the user base or traffic on our platform grows.
12. Contextual adaptability	Our software allows for highly tailorable and versatile EMA and EMI designs in terms of item content, survey flow and notification set-up. Unsupported features or functionalities can be developed against a development cost. The app is available in more than 15 languages.
13. Replicability and documentation	There is an online manual with up-to-date software documentation (see www.m-Path.io/manual) and a moderated help centre (see http://www.reddit.com/r/mpath).
14. Data security	Identifiable participant/patient information is not required to register to the mobile app. Server-phone communication is end-to-end encrypted. m-Path's source code is obfuscated, limiting the exploitation of unforeseen vulnerabilities. Researcher/clinicians accounts are supplied with two-factor authentication. Physical servers are only accessible by maintenance personnel.
15. Compliance with national guidelines	m-Path complies with the European Union's GDPR policy, one of the strictest privacy policies applied in the world, meaning that participants have full control over their data. The prerequisites for medical device certification are being investigated.
16. Fidelity of intervention	Various real-time graphs in the online dashboard visualize participants’/patients’ responses and engagement (e.g., compliance rates). Adherence can be boosted *via* our award system (e.g., gamification).

## Data Availability

The original contributions presented in the study are included in the article/Supplementary Material, further inquiries can be directed to the corresponding author.

## Footnotes

^1^Because the field is constantly developing, we refrain from an explicit comparison with other existing platforms, as such an attempt would become readily outdated and not be comprehensive. Nevertheless, for two dynamic, crowd-sourced databases that provide a comparative overview of existing EMA / EMI / sensing solutions, see here and here.

^2^The current overview is based on the most recent software versions at the time of publication (i.e., version 14b of the researcher’s dashboard, and version 289 [3.12.19] and 2.8.0 of the Android and iOS app, respectively). Although we aimed to keep our workflow description version non-specific, minor adaptations in future set-ups cannot be ruled out.

^3^Note that m-Path does not provide offline sampling schemes that deliver notifications when participants do not have an active internet connection (e.g., based on a predefined trigger template that is downloaded in advance). The reason for this decision is that it becomes increasingly difficult to guarantee that offline sampling is supported across the entire ecology of mobile devices. That is, for a growing number of phones, applications that run in the background are automatically killed when a person does not interact with them on a regular basis (as a battery saving strategy). When the background app is killed, it cannot send notifications anymore. This makes it hard to reliably push notifications with template-based sampling apps for all mobile device types. Simultaneously, the prevalence and accessibility of mobile internet connections are on the rise, diminishing the significance of needing an active internet connection as a potential obstacle.

^4^Because Google banned Huawei from using its Android operating system since 2019 (46), m-Path (currently) does not work on the latest Huawei phones (Mate 30 series or newer).
